# The Evidence That 25(OH)D3 and VK2 MK-7 Vitamins Influence the Proliferative Potential and Gene Expression Profiles of Multiple Myeloma Cells and the Development of Resistance to Bortezomib

**DOI:** 10.3390/nu14235190

**Published:** 2022-12-06

**Authors:** Karolina Łuczkowska, Piotr Kulig, Bartłomiej Baumert, Bogusław Machaliński

**Affiliations:** 1 Department of General Pathology, Pomeranian Medical University, 70-111 Szczecin, Poland; 2 Department of Hematology and Transplantology, Pomeranian Medical University, 71-252 Szczecin, Poland

**Keywords:** multiple myeloma, vitamin D, 25(OH)D3, vitamin K, VK2 MK-7, resistance to bortezomib

## Abstract

Multiple myeloma (MM) remains an incurable hematological malignancy. Bortezomib (BTZ) is a proteasome inhibitor widely used in MM therapy whose potent activity is often hampered by the development of resistance. The immune system is vital in the pathophysiology of BTZ resistance. Vitamins D (VD) and K (VK) modulate the immune system; therefore, they are potentially beneficial in MM. The aim of the study was to evaluate the effect of BTZ therapy and VD and VK supplementation on the proliferation potential and gene expression profiles of MM cells in terms of the development of BTZ resistance. The U266 MM cell line was incubated three times with BTZ, VD and VK at different timepoints. Then, proliferation assays, RNA sequencing and bioinformatics analysis were performed. We showed BTZ resistance to be mediated by processes related to ATP metabolism and oxidative phosphorylation. The upregulation of genes from the SNORDs family suggests the involvement of epigenetic mechanisms. Supplementation with VD and VK reduced the proliferation of MM cells in both the non-BTZ-resistant and BTZ-resistant phenotypes. VD and VK, by restoring proper metabolism, may have overcome resistance to BTZ in vitro. This observation forms the basis for further clinical trials evaluating VD and VK as potential adjuvant therapies for MM patients.

## 1. Introduction

Multiple myeloma (MM) is a plasma cell malignancy that accounts for approximately 1–2% of all neoplasms [1,2,3] and 10% of hematological malignancies [2]. Initially, it was considered a highly aggressive disease with a relatively poor prognosis. However, over time, patients’ outcomes improved significantly through the development and implementation of novel therapies [4]. There were several turning points in MM therapy. Of particular importance was the development of bortezomib (BTZ) and its wide implementation to chemotherapy regimens. BTZ is a proteasome inhibitor [5]. More precisely, it acts as an inhibitor of the 26S proteasome, a major regulator of intracellular protein degradation. BTZ selectively and reversibly inhibits the chymotryptic site. This function allows BTZ to inhibit the degradation of proteins critically involved in the regulation of cell proliferation and survival, ultimately leading to growth inhibition and apoptosis [6].

Despite the potent anti-MM activity of BTZ, over time, there is a subset of patients who become refractory to BTZ. This phenomenon is one of the greatest challenges in the management and treatment of MM. Chemotherapy exerts enormous selective pressure on malignant myeloma cells. Under such circumstances, the drug-resistant phenotype becomes favorable and may eventually regain control, leading to relapse or disease progression. The underlying molecular mechanisms are not entirely elucidated and appear to be multifactorial. For instance, chromosomal instability (CIN) was demonstrated to be involved in BTZ resistance in MM. NEK2 is the CIN gene that was highly expressed, and its overexpression was associated with poor clinical outcomes. Moreover, its increased expression promoted MM cell proliferation and drug resistance, predominantly mediated via upregulation of efflux pumps [7] and autophagy [8]. Interestingly, NEK2 inhibitors were shown to overcome BTZ resistance in xenograft myeloma mouse models [9]. Simultaneously, disrupted lipid metabolism, with a particular emphasis on the elongation of very long chain fatty acids protein 6 (ELOVL6), may also contribute to the emergence of the resistant phenotype [10]. The immune system plays a pivotal role in myelomagenesis [11], but the prevalence of pro-inflammatory processes may also contribute to BTZ resistance. Beyar-Katz et al. demonstrated that pro-inflammatory macrophages exposed to BTZ promote the enrichment of MM-tumor-initiating cells (MM-TIC). This process was regulated by IL-1. The study concluded that pro-inflammatory macrophages in bone marrow biopsies represent a potential prognostic biomarker for acquired MM resistance to BTZ therapy [12]. It was also established that B-cell activating factor (BAFF) and its receptors played a functional role in the macrophage-mediated resistance of MM cells to BTZ through the activation of both classical and alternative NF-κB pathways [13].

As stated above, altered immune system function combined with the tumor microenvironment contributes to the development of resistance to BTZ. Moreover, the interplay between bone marrow stromal cells (BMSCs) and MM cells may enhance these effects [14] and consequently contribute to myelomagenesis, disease progression and promotion of the refractory phenotype of MM cells. Therefore, it can be hypothesized that easy-to-implement adjuvant therapies aimed at restoring proper immune system activity may improve patient outcomes. One of the potential adjuvant therapies in MM may be vitamin D (VD) and vitamin K (VK) supplementation. These vitamins, especially VD, exert multiple so-called non-classical actions that are predominantly associated with immune system modulation. 25(OH)D3 is more stable than the 1.25(OH)D3 isoform [15], and it was demonstrated that repletion of 25(OH)D3 exerts metabolic advantages over repletion of 1.25(OH)2D3 via multiple cellular mechanisms [16]. This applies to both innate and adaptive immunity as the VD receptor (VDR) is present in multiple subpopulations of immune cells [17,18]. Thus, numerous immune processes and pathways are under VD influence. VD deficiency is associated with respiratory tract infections, autoimmunity [19] and poorer outcomes in some cardiovascular diseases [20]. On the other hand, VD exerts immunomodulatory and anti-inflammatory actions [21]. This effect should be thoroughly investigated, especially in the case of cancers, as the malignancy is associated with an inflammatory response [22]. Hence, VD may be potentially beneficial in malignant neoplasms. VD has been found to regulate the proliferation, differentiation and apoptosis of many types of cancer cells [23].

In addition to VD, VK, with a particular emphasis on the VK2 isoform, exhibits non-classical actions that may improve the management and treatment of MM. VK2 controls the activity of extrahepatic vitamin K-dependent proteins; hence, it is responsible for a majority of the non-classical actions, while the VK2 MK-7 isoform is the most readily absorbed [24]. Several studies have shown promising results. First of all, it is necessary to mention the influence of VK on the immune system, which manifests itself in its anti-inflammatory properties [25,26,27]. On top of that, it was established that VK exhibits anti-cancer activity, which applies to both non-hematological [28,29,30] and hematological malignancies [31,32,33]. Importantly, similar effects were observed in MM cell lines [34]. Apart from VK, its analogs also exhibited anti-MM actions [35]. It is believed that both vitamins should be supplemented simultaneously [36]. Accordingly, it can be hypothesized that VD and VK can mutually potentiate each other’s effect, and their parallel administration can be beneficial in MM. Anti-MM properties of VD were demonstrated predominantly in in vitro studies [37,38]. Additionally, there were conducted human studies, both clinical trials and correlational, revealing the role of VD in MM patients [39,40,41].

The aim of the study was to evaluate the influence of BTZ therapy, VD and VK supplementation and concurrent therapy with BTZ, VD and VK on: (i) the proliferative potential of MM cells, (ii) gene expression profiles of MM cells and (iii) biological processes involved in the development of BTZ resistance.

## 2. Materials and Methods

### 2.1. Cell Culture

The human multiple myeloma cell line U266 (ATCC, Manassas, VA, USA) was applied in this study. U266 cells were incubated at a density of 1 × 10^5^–1 × 10^6^ cells/mL in T-75 flasks with RPMI-1640 Medium (ATCC, Manassas, VA, USA) modified to contain 2 mM L-glutamine, 10 mM HEPES, 1 mM sodium pyruvate, 4500 mg/L glucose, 1500 mg/L sodium bicarbonate, penicillin (100 U/mL) and streptomycin (100 µg/mL) and with 15% fetal bovine serum at 37 °C in a saturated, humid atmosphere containing 5% CO_2_. The medium was changed every two or three days.

The mouse fibroblast cell line L929 (ATCC, Manassas, VA, USA) was used in this study to evaluate the effect of the applied dose of vitamin D (10^−6^ M) on healthy, non-cancerous cells. L929 cells were incubated in T-75 flasks until a confluence of up to 80% was obtained with Eagle’s Minimum Essential Medium modified to contain Earle’s Balanced Salt Solution (ATCC, Manassas, VA, USA), non-essential amino acids, 2 mM L-glutamine, 1 mM sodium pyruvate, 1500 mg/L sodium bicarbonate, penicillin (100 U/mL) and streptomycin (100 µg/mL) and with 10% fetal bovine serum at 37 °C in a saturated, humid atmosphere containing 5% CO_2_. The medium was changed every two or three days.

### 2.2. RNA Isolation

Total RNA was isolated from three separate cell incubations for all groups. Additionally, 1 × 10^6^ U266 cells were used each time to isolate one RNA sample. The applied isolation kit (mirVana, miRNA Isolation Kit, ThermoFisher, Waltham, MA, USA) allows the simultaneous isolation of total RNA enriched with miRNA molecules. Total RNA isolation was performed according to the manufacturer’s protocol. The concentration and quality of the isolated RNA were measured using TapeStation 4510 (Agilent Technologies, Santa Clara, CA, USA). The Agilent RNA ScreenTape System kit was used for measurement (Agilent Technologies, Santa Clara, CA, USA). All samples showed RINs ≥9, which confirms the high quality of the genetic material.

### 2.3. Proliferation Assay

Assessment of proliferation was performed after each treatment with BTZ and vitamins. The Alamar blue assay (ThermoFisher, Waltham, MA, USA) was selected for the assessment of proliferation, which reduces resazurin (a blue non-fluorescent dye) to resorufin (a red fluorescent dye). The amount of resorufin is proportional to the number of cells. Fluorescence measurement was performed with the Varioskan LUX apparatus (ThermoFisher, Waltham, MA, USA) at the Ex560/Em590 wavelength. Measurements were performed on days 1, 3, 6, 8 and 10 of cell culture after each treatment. Cells were incubated at a density of 1 × 10^4^ cells/well (U266) or 1 × 10^3^ cells/well (fibroblast) in 96-well culture plates. Each study group and control were seeded in 8 separate wells for each measurement day.

### 2.4. The Course of the Experiment and Determination of the Dose of BTZ and Vitamins

**Figure 1:**
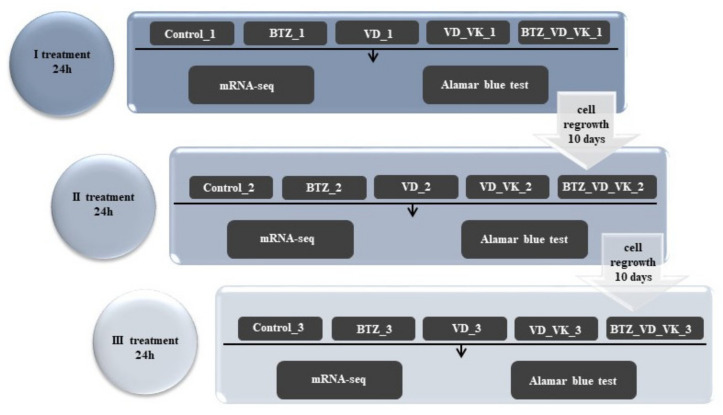
Scheme of study.

U266 cells were treated three times with BTZ (2.75 nM) (Cell Signaling Technology, Danvers, MA, USA), vitamins: 25(OH)D3 (VD) (10^−6^ M) (Sigma-Aldrich, Saint Louis, MO, USA) and K2MK7 (VK) (10^−5^ M) (Sigma-Aldrich, Saint Louis, MO, USA) for 24 h with 10-day intervals between treatments. After each treatment with BTZ or vitamins, genetic material was isolated, Alamar blue assay was performed, and 1 × 10^6^ cells were allowed to proliferate further in BTZ and vitamin-free medium. There were 4 study groups (VD—treated with vitamin 25(OH)D3; BTZ—treated with bortezomib; VD_VK—treated simultaneously with vitamin 25(OH)D3 and K2MK7; BTZ_VD_VK—treated simultaneously with bortezomib, vitamin 25(OH)D3 and K2MK7) and untreated control cells. The study was carried out in three technical repetitions for each group. The study scheme is shown in Figure 1.

The BTZ dose was determined based on the Alamar blue test by treating U266 cells with different doses of BTZ for 24 h. The selected dose (2.75 nM, *p* < 0.0001) after the first treatment induced the death of more than 50% of U266 cells. Subsequent treatments resulted in the development of resistance to BTZ.

The aim of the study was to select a dose of vitamin D that would simultaneously inhibit cancer cells and not inhibit the growth of non-cancerous cells. The dose of vitamin D was determined on the basis of literature data [42,43] and our own experience. After administration of several doses of vitamin D, phosphorylation of the vitamin D receptor (VDR) on the surface of U266 cells was investigated by Western blot. The highest degree of phosphorylation was obtained after 24 h treatment of cells with a dose of 10^−6^ M (data not shown). Additionally, in order to prove the correctness of the selected dose of vitamin D, the Alamar blue test was performed on non-cancerous cells—human fibroblasts. Cells were treated three times with 10^−6^ M vitamin 25(OH)D3 at 10-day intervals. Proliferation was tested on days 1, 3, 6, 8 and 10 of culture. The vitamin K dose was established on the basis of literature data [34].

### 2.5. RNA-Seq

The Illumina Stranded mRNA Prep kit (Illumina, San Diego, CA, USA) and IDT for Illumina RNA UD Indexes Set A, Ligation (Illumina, San Diego, CA, USA) were used to prepare the sequencing library, and all procedures were performed according to the manufacturer’s protocol. Briefly, 1000 ng of RNA from each sample was used to create the library. The first step was to purify the material with oligo (dT) magnetic beads, which capture mRNA with polyA tails, and to fragment mRNA to prepare the material for cDNA synthesis. In the next step, reverse transcription was performed to generate the cDNA strand. The First Strand Synthesis Mix contained Actinomycin D, which enables RNA-dependent synthesis and improves strand specificity while preventing false DNA-dependent synthesis. The next steps were the synthesis of the blunt-ended second strand, the addition of adenine nucleotides (A) to the 3’ ends of the blunt fragments to prevent them from ligating together during adapter ligation and pre-index anchor ligation. After purification of the libraries with the AMPure XP kit (Beckman Coulter Diagnostics, Brea, CA, USA), indexes and primer sequences were added to form clusters during sequencing. The libraries were then amplified (10 cycles containing: 98 °C for 10 s, 60 °C for 30 s and 72 °C for 30 s). After amplification, the libraries were purified with the AMPure XP kit, and their purity and molarity were measured using a TapeStation (Agilent, Santa Clara, CA, USA) apparatus and an Agilent D1000 ScreenTape Assay kit (Agilent, Santa Clara, CA, USA). Subsequently, each library was diluted to 1.3 nM, and all libraries were pooled and denatured with 0.2 N NaOH. The final loading library concentration was 1.3 pM. Sequencing was performed on the NextSeq 550 instrument (Illumina, San Diego, CA, USA) using NextSeq 500/550 High Output Kit v2.5 (150 cycles) reagents.

### 2.6. Bioinformatic Analysis

Demultiplexing of the sequencing reads was carried out using Illumina bcl2fastq (2.20), which led to obtaining FASTQ files. Sequencing quality was controlled using the FastQC tool [44]. Adapters and low-quality sequences were trimmed with Cutadapt [45]. Trimmed raw reads were aligned to the human reference genome (hg 19) with the relevant GTF file downloaded from the Ensembl database. Alignment was carried out using the free Spliced Transcripts Alignment to a Reference (STAR) (version 2.5.2b) software by Alexander Dobin from Cold Spring Harbor Laboratory, Cold Spring Harbor, NY, USA [46]. The overall summary results, including the number of successfully assigned reads with unnormalized counts, were obtained using featureCounts [47]. Row counts and gene length data were downloaded to “R” for transcript per million (TPM) normalization. Genes with low median counts for all samples were removed from the final count table. For all genes, the fold change was calculated by dividing the normalized TPM value for the study group compared to the TPM of the control group. Genes with a fold change of more than 2 or less than −2 were considered as differentially expressed genes (DEGs).

### 2.7. Statistical Methods

Arithmetic means and standard deviations (SDs) were calculated using MS Excel. The comparison of parameters between the two groups was performed using the Student’s *t*-test, and a *p*-value of <0.05 was considered statistically significant.

## 3. Results

The research aimed to broaden knowledge in three specific areas. The first one was to identify and understand the molecular factors responsible for the development of resistance to BTZ in myeloma cells. The second area concerns the role of vitamins D and K in the development of cancerous myeloma cells. The third attempt was to understand the influence of vitamin D and K on the development of resistance to BTZ in myeloma cells. The results are presented in such a way as to clearly present the three key assumptions of the work.

### 3.1. Proliferation Assay

A proliferation assay was performed after each treatment/incubation on days 1, 3, 6, 8 and 10 of cell culture (as described in Section 2.3 Proliferation assay). Cells were incubated for 4 h with resazurin.

#### 3.1.1. U266 Myeloma Cells

##### Changes in Proliferation Resulting from the Development of Resistance of U266 Myeloma Cells to BTZ

**Figure 2:**
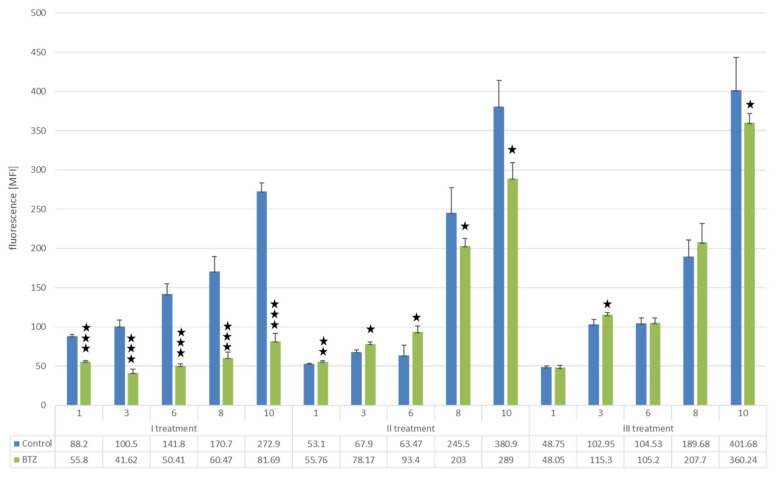
Analysis of U266 cell proliferation using the Alamar blue assay. The scheme shows the results after each treatment with BTZ or placebo (control). Measurements were taken 1, 3, 6, 8 and 10 days after treatment with the agents. Student’s *t*-test; *** *p* < 0.000001; ** *p* < 0.0001; * *p* < 0.05.

After the first treatment with BTZ on day 10 of cell culture, a reduction in proliferation of 70.06% was observed compared to control cells (*p* < 0.0001). After the second treatment with BTZ, there was a decrease in proliferation by 24.12% (*p* < 0.0001) compared to the control cells, while after the third BTZ treatment, proliferation only decreased by 10.31% (*p* < 0.01). This study confirmed the development of the BTZ-resistant phenotype of U266 myeloma cells (Figure 2).

##### Effect of Vitamins D and K on Proliferation of U266 Myeloma Cells

**Figure 3:**
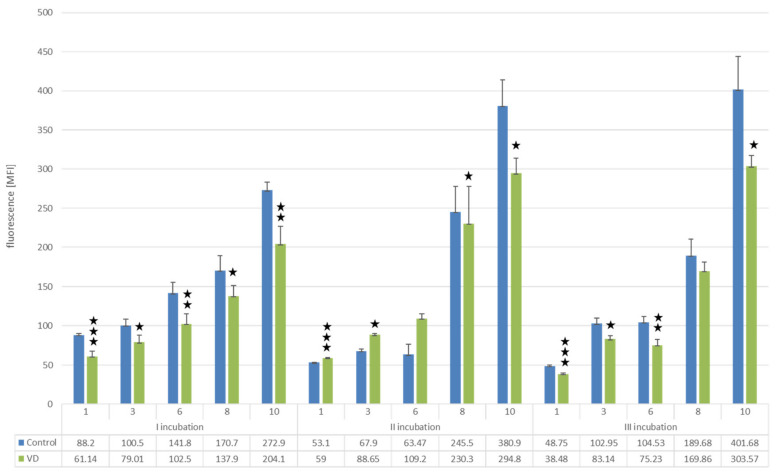
Analysis of U266 cell proliferation using the Alamar blue assay. The diagram shows the results after incubating three times every 10 days with 25(OH)D3 or placebo (control). Measurements were performed on days 1, 3, 6, 8 and 10 after each incubation. Student’s *t*-test; *** *p* < 0.000001; ** *p* < 0.0001; * *p* < 0.05.

The addition of 25(OH)D3 to the culture reduced the proliferation of myeloma cells after each incubation by more than 20% compared to control (on day 10 of cell culture: first incubation 25.21%, *p* < 0.001; second incubation 22.60%, *p* < 0.001; third incubation 24.42%, *p* < 0.0001) (Figure 3).

**Figure 4:**
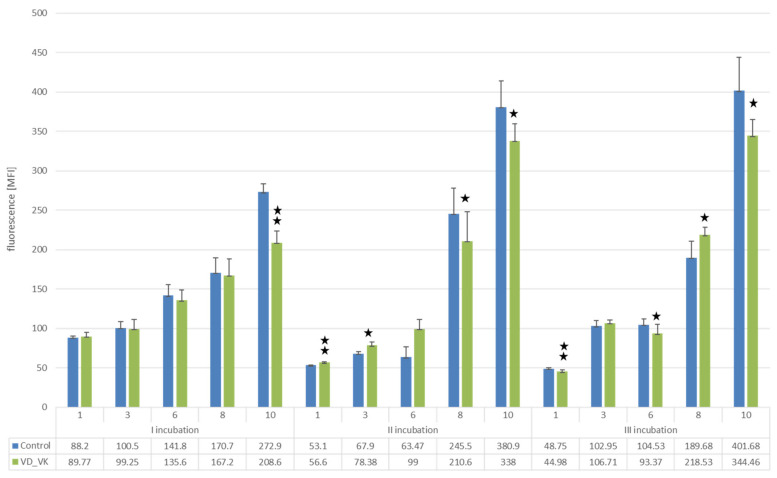
Analysis of U266 cell proliferation using the Alamar blue assay. The diagram shows the results after three times simultaneous incubation every 10 days with VD and VK or placebo (control). Measurements were performed on days 1, 3, 6, 8 and 10 after each incubation. Student’s *t*-test; ** *p* < 0.0001; * *p* < 0.05.

The simultaneous incubation of U266 cells with 25(OH)D3 and K2MK7 showed a similar effect after the first application and slightly less pronounced effects after the second and third vitamin applications (Figure 4).

##### Changes in the Proliferation Level of U266 Myeloma Cells with a BTZ-Resistant Phenotype Induced by Vitamin D and K

**Figure 5:**
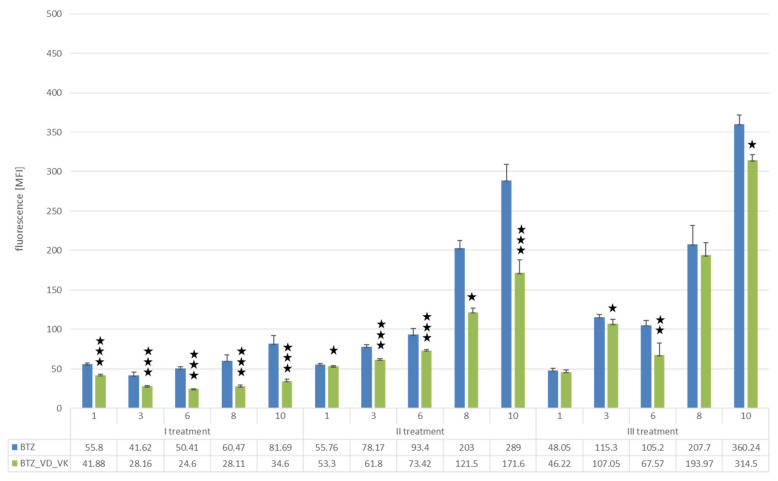
Analysis of U266 cell proliferation using the Alamar blue assay. The diagram shows the results after each BTZ treatment with simultaneous VD and VK incubation or BTZ alone (control). Measurements were performed on days 1, 3, 6, 8 and 10 after treatment with the agents. Student’s *t*-test; *** *p* < 0.000001; ** *p* < 0.0001; * *p* < 0.05.

After the first treatment of cells with both BTZ and vitamins, a reduction in proliferation of 57.64% (*p* < 0.00001) was observed on day 10 compared to cells treated with BTZ alone. In the non-BTZ-resistant line, vitamins additionally reduced the level of proliferation. After the second treatment with BTZ and vitamins (BTZ-resistant cell line), a decrease in proliferation of 40.62% (*p* < 0.00001) was observed relative to the BTZ-treated cells. The vitamins significantly reduced the level of myeloma cell proliferation in the BTZ-resistant U266 cell line. However, after the third treatment with BTZ and vitamins, a decrease in proliferation was only observed by 12.69% (*p* < 0.001) compared to the cells treated with BTZ alone (Figure 5).

#### 3.1.2. L929 Fibroblast Cells

The proliferation analysis showed no effect of 25(OH)D3 at a dose of 10^−6^ M on fibroblast cells. After 10 days of culture, the results were very similar in both VD-treated and control cells or even higher after treatment with VD (I incubation: VD 148.35 ± 12.28; control 129.74 ± 11.40, *p* = 0.04; II incubation: VD 91.156 ± 11.77; control 78.39 ± 2.59, *p* = 0.02; III incubation: VD 76.35 ± 11.36; control 60.88 ± 8.74, *p* = 0.01). The obtained results confirm the selection of the appropriate dose of VD, which at the same time has no toxic effect on healthy, non-cancerous cells and inhibits the proliferation of neoplastic cells.

### 3.2. mRNA-Seq

**Figure 6:**
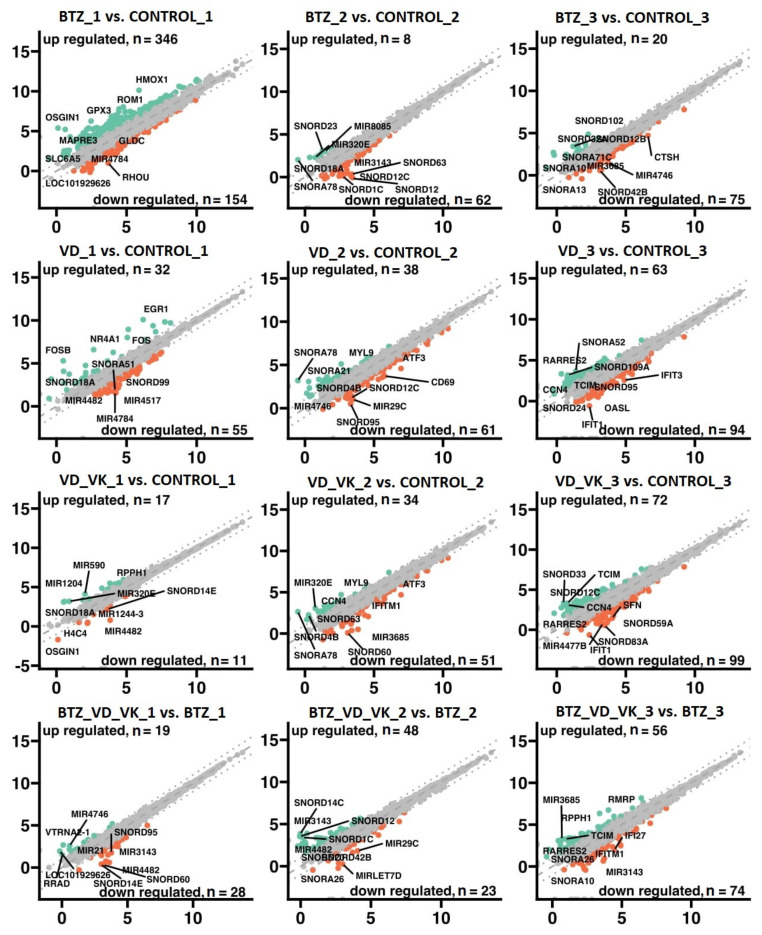
Gene expression profiles obtained in bioinformatics analysis of U266 myeloma cells after treatment with BTZ, VD and VK. Gray dots show genes with a fold <2 or *p* > 0.05 cutoff from the analysis. The red dots show the genes with decreased expression compared to the control, while the green dots show the genes with increased expression compared to the control. The graphs show the symbols of the most altered genes.

The transcriptome profiles for individual groups are presented in a volcano plot (Figure 6). The criteria of gene classification were *p* < 0.05 and fold ≥2. The highest number of genes with altered expression was obtained in the comparisons of BTZ_1 and Control_1 (346 upregulated and 154 downregulated genes); BTZ_VD_VK_1 and Control_1 (245 genes upregulated and 65 downregulated); VD_VK_3 and Control_3 (72 genes upregulated and 99 downregulated); and BTZ_VD_VK_3 and BTZ_3 (56 genes upregulated and 74 downregulated).

As part of the bioinformatic analysis, functional analysis of the annotation of genes with differentiated expression was performed using the bioinformatics tool Database for Annotation, Visualization and Integrated Discovery (DAVID) with the GO BP Direct database and Gene Set Enrichment Analysis (GSEA).

**Figure 7:**
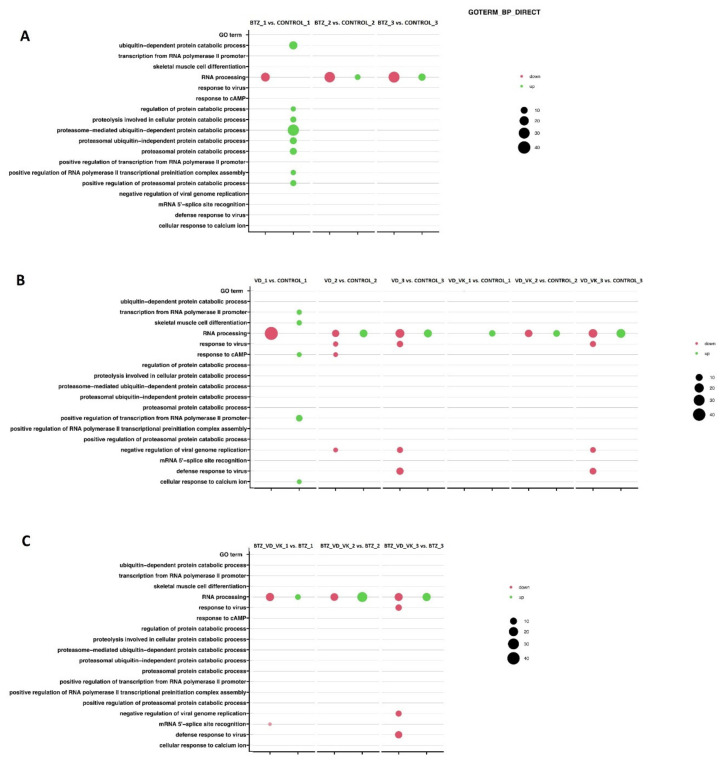
The bubble plot shows the biological processes involved in the development of BTZ resistance, the effects of VD and VK on myeloma cells and their role in the development of resistance to BTZ. The size of the bubble is proportional to the number of genes involved in the process. The red color of the bubble means inhibition of the biological process, and green means its increase (*p* < 0.05, fold ≥2). Part of diagram (**A**) shows the processes after treatment with BTZ vs. control; (**B**) shows the processes after incubation with VD_VK vs. control; (**C**) shows the processes after treatment with BTZ_VD_VK vs. BTZ.

DAVID is a functional annotation and enrichment analysis. The results obtained from this analysis are shown in the bubble plot (Figure 7). The following criteria were used to create the plot: *p* < 0.05, fold ≥ 2, adjusted method = Benjamini and the minimum number of genes to generate a bubble = 5. Each bubble represents a separate biological process according to the Gene Ontology (GO) classification, and its weight is proportional to the number of genes involved in the process. GSEA is a bioinformatics tool that analyzes gene groups and their involvement in biological processes and signaling, without focusing on individual genes.

#### 3.2.1. Transcriptional Changes Associated with the Development of Resistance of U266 Myeloma Cells to BTZ

During the experiment, U266 myeloma lines resistant to BTZ were obtained. Analysis of the transcriptome revealed that several important biological processes were responsible for its development. One of the most important observations was the involvement of the “RNA processing” process (Figure 7A). Genes from the small nucleolar RNAs, C/D box (SNORDs) family, which are responsible for changes in DNA methylation, were involved in this biological process. After the first BTZ treatment, inhibition of the SNORDs genes was observed, while after the subsequent treatments, when the BTZ-resistant phenotype was obtained, some genes from this family were upregulated (Table S1 in Supplementary Materials). Changes in the expression of these genes clearly indicate the influence of epigenetic changes on the development of resistance to BTZ.

**Figure 8:**
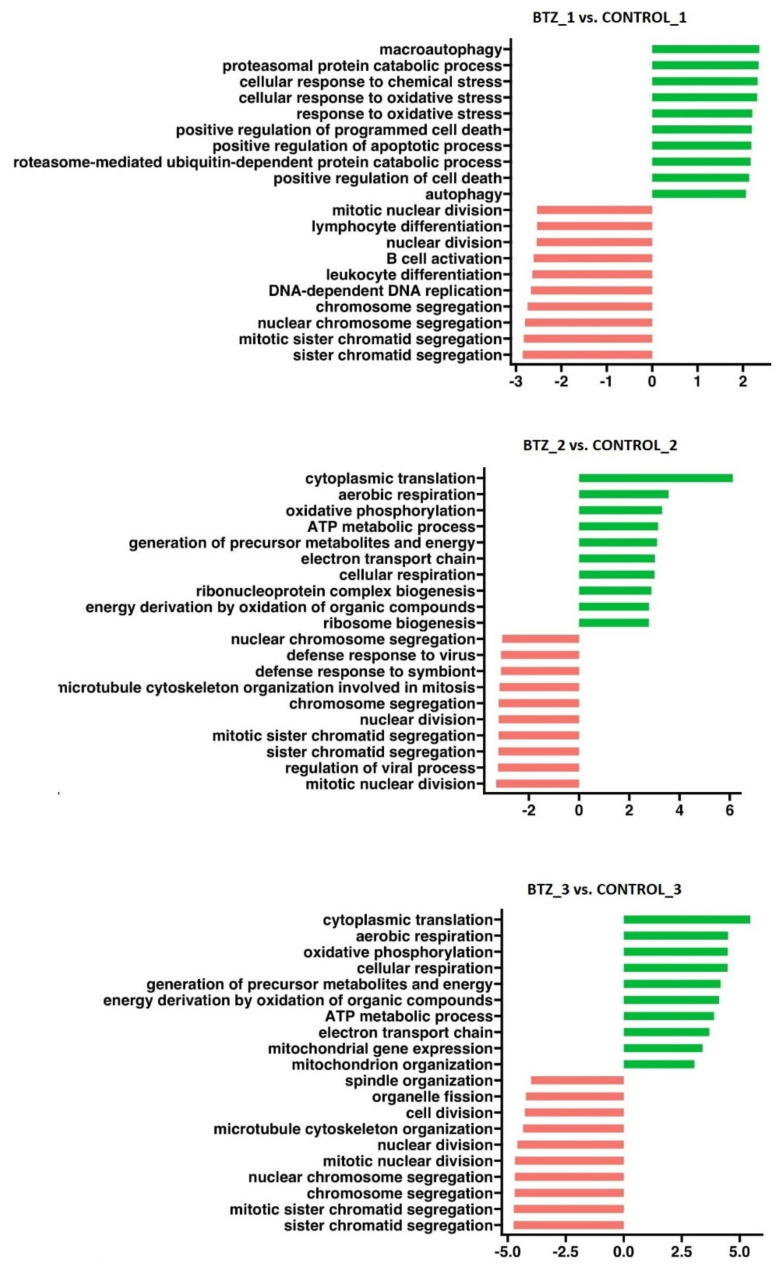
GSEA performed in U266 cells after BTZ administration (BTZ_1—first treatment; BTZ_2—second treatment; BTZ_3—third treatment). The plots show the normalized enrichment scores (NES) for the most positively and negatively enriched gene sets. The green color shows activation of selected processes, and the red color shows inhibition. *p* < 0.05.

After the first treatment with BTZ (cells not yet resistant to BTZ), GSEA analysis (Figure 8) showed activation of processes such as cellular response to oxidative stress, positive regulation of programmed cell death and positive regulation of the apoptotic process and inhibition of processes such as mitotic nuclear division, mitotic sister chromatid segregation and nuclear chromosome segregation. The second treatment of BTZ cells, which resulted in a BTZ-resistant phenotype, triggered the activation of processes (Figure 8) such as oxidative phosphorylation, the generation of precursor metabolites and energy, the ATP metabolic process, cellular respiration and energy derivation by oxidation of organic compounds, which may be of key importance in the development of resistance to BTZ. After the third treatment with BTZ cells, activation of similar biological processes was observed as after the second treatment but with further intensification (Table 1).

#### 3.2.2. Effect of VD and VK on Transcriptional Changes in Myeloma Cells U266

After the first incubation of cells with VD, DAVID analysis (Figure 7B) showed a reduction in “RNA processing” and an increase in processes such as transcription from RNA polymerase II promote, response to cAMP, positive regulation of transcription from RNA polymerase II promote and cellular response to calcium ions. After the second VD incubation, it was observed that the expression of some genes from “RNA processing” was increased. Moreover, it was observed that the “response to cAMP” process, which was upregulated after the first administration, was inhibited after the second application. After the third incubation with VD, similar changes were observed as after the second procedure.

After the first incubation of cells with both VD and VK, DAVID analysis showed only an increase in “RNA processing”. The second and third administrations of vitamins showed the activation and inhibition of practically the same processes as when using VD alone (Figure 7B).

**Figure 9:**
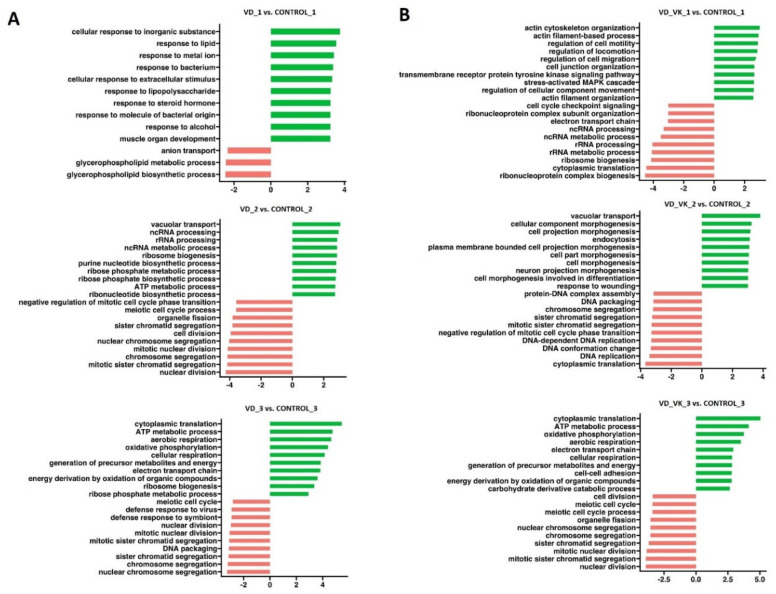
GSEA performed in U266 cells after VD (**A**) or VD and VK (**B**) administration (VD_1 or VD_VK_1—first incubation; VD_2 or VD_VK_2—second incubation; VD_3 or VD_VK_3—third incubation). Plots show normalized enrichment scores (NES) for the most positively and negatively enriched gene sets. The green color shows activation of selected processes, and the red color shows inhibition. *p* < 0.05.

GSEA analysis (Figure 9A) showed after the first incubation with VD the inhibition of processes such as the glycerolipid metabolic process, anion transport, the glycerophospholipid metabolic process and the glycerophospholipid biosynthetic process and activation of processes such as cellular response to extracellular stimulus, hemopoiesis and the positive regulation of immune system processes. After the second incubation with VD, activation of the following processes was demonstrated: vacuolar transport, ncRNA processing, rRNA processing and the ATP metabolic process, as well as inhibition of processes related mainly to cell division (meiotic cell cycle process, cell division, chromosome segregation and nuclear division). After the third incubation, a significant intensification of the “ATP metabolic process” was observed in relation to its level after the second application (II incubation NES = 2.77, *p* < 0.0001; III incubation NES = 4.80, *p* < 0.0001). Moreover, processes such as aerobic respiration, oxidative phosphorylation, the generation of precursor metabolites and the energy process were activated. In contrast, we observed inhibited DNA packaging, nuclear division and nuclear chromosome segregation.

At the same time, GSEA analysis showed after the first incubation with VD and VK (Figure 9B) the inhibition of processes such as the ATP metabolic process, cell cycle checkpoint signaling and rRNA processing and activation of processes such as regulation of cell motility, cell junction organization and stress-activated mitogen-activated protein kinases (MAPK) cascade. After the second administration of VD and VK, activation of the following processes was demonstrated: vacuolar transport and plasma membrane bounded cell projection morphogenesis, as well as inhibition of processes such as DNA packaging, sister chromatid segregation and DNA replication. After the third incubation, an increase in processes such as the ATP metabolic process, aerobic respiration and energy derivation by oxidation of organic compounds was observed. Processes such as meiotic cell cycle, sister chromatid segregation and nuclear division were inhibited. In conclusion, after the third treatment of cells with VD alone versus treatment with VD and VK, very similar gene expression results were obtained.

#### 3.2.3. Transcriptional Changes Induced by VD and VK in U266 Myeloma Cells in BTZ-Resistant Phenotype

DAVID analysis (Figure 7C) after the first treatment with BTZ and incubation with vitamins (non-resistant phenotype of cells) showed inhibition and activation of the “RNA processing” process. Six genes regulating this process were upregulated (*SNORD19B, SNORD42B, SNORA75, SNORD1C, SNORD69* and *SNORD75*), and fifteen were downregulated (*SNORD116-20, SNORD116-21, SNORD116-22, SNORD60, SNORD59A, SNORD48, SNORA21, SNORD95, SNORA26, SNORA80E, SNORA51, SNORD1A, SNORD12, SNORD17* and *SNORD97*). After the second treatment (BTZ-resistant phenotype of cells), the same situation was observed, but the process was intensified. In the “RNA processing” process, 24 upregulated genes emerged, and 13 genes lowered their expression. After the third treatment of the cells, an up- and downregulated “RNA processing” process was also observed (15 genes upregulated and 14 downregulated). However, there were also additional downregulated processes such as the defense response to viruses and negative regulation of viral genome replication.

**Figure 10:**
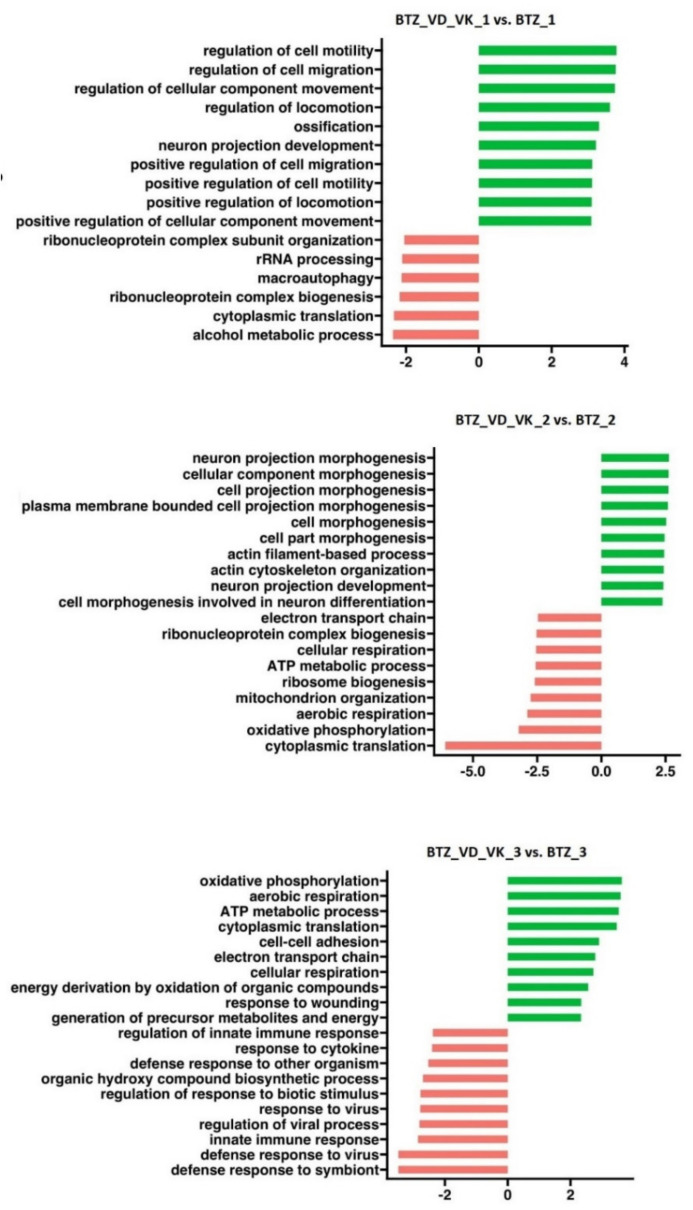
GSEA performed in U266 cells after BTZ and vitamin administration (BTZ_VD_VK_1—first treatment; BTZ_VD_VK_2—second treatment; BTZ_VD_VK_3—third treatment). Plots show normalized enrichment scores (NES) for the most positively and negatively enriched gene sets. The green color shows activation of selected processes, and the red color shows inhibition. *p* < 0.05.

After the first treatment with BTZ and vitamins, GSEA analysis (Figure 10) showed inhibition of processes such as the rRNA metabolic process, ribonucleoprotein complex biogenesis and cytoplasmic translation and activation of processes such as positive regulation of cell death, regulation of cell motility and regulation of cell migration. After the second treatment in the BTZ-resistant myeloma cell line, the most important observation seems to be the inhibition of energy-related processes such as the ATP metabolic process, aerobic respiration and oxidative phosphorylation, the activation of which was observed in BTZ-resistant cells. The processes related to the increase in cellular energy may be significantly related to the development of resistance to BTZ, and the use of VD and VK may limit the development of these processes.

However, after the third treatment of BTZ-resistant cells with BTZ and vitamins, a drastic change from the results obtained after the second treatment was observed. The processes related to energy production were activated (Figure 10).

## 4. Discussion

VD acts through VDR, which is present in multiple tissues and cell types (18) including malignant MM cells [48]. Therefore, it can be assumed that VD also affects neoplastic cells. In our study, we first incubated MM U266 cells with BTZ. After the second passage, we obtained a BTZ-resistant phenotype. Subsequently, we demonstrated that adding 25(OH)D3 alone to the medium in which MM cells were incubated decreased their proliferation. We then examined whether the combination of 25(OH)D3, VK2 MK-7 and BTZ could exert a beneficial effect. Interestingly, cells treated with the above-mentioned combination of molecules inhibited the development of resistance to BTZ, i.e., their proliferation was reduced compared to cells incubated with BTZ alone after the second and third passages. In addition, vitamins significantly reduced proliferation in the non-BTZ-resistant myeloma cell line, which may also positively affect the outcomes of MM patients.

The effects of VD alone and in combination with anti-MM drugs have previously been studied in vitro and in vivo. Busch et al. investigated whether the immunomodulatory drug lenalidomide improved the anti-MM activity of myeloma-associated macrophages (MAMs) elicited by MOR202 (monoclonal antibody anti-CD38) and whether the VD pathway was a part of its modulating effect. First, they established that MAMs of MM patients exhibit a defective VD pathway and that lenalidomide restores VD metabolism to MAMs. Moreover, they demonstrated that activation of the VD pathway enhances the tumoricidal effector mechanisms of macrophages. Subsequently, they found that 1.25(OH)D3 enhances CD38 expression on MM cells, i.e., target cells for MOR202. Since the efficacy of monoclonal antibodies is partially dependent on target antigen expression, when incubated with 1.25(OH)D3, MOR202 has a higher propensity to bind to the surface of MM cells. The study concluded that the combination of lenalidomide and exogenously added 25(OH)D3 (then converted to 1.25(OH)D3 by the macrophages) improves the efficacy of MOR202-mediated cytotoxicity of MAMs. MAMs have the ability to kill MM cells, predominantly via antibody-dependent cellular phagocytosis (ADCP), treated with MOR202. The activation of the VD pathway by lenalidomide as well as the concurrent supplementation of VD enhances the therapeutic efficacy of MOR202. The obtained results suggest that maintaining proper 25(OH)D3 concentrations in MM patients may be of clinical significance [37]. Another study investigated the antiproliferative effects of 1.24(OH)2D2 in combination with bisphosphonate pamidronate on an MM H929 cell line. It was established that 1.24 (OH)2D2 alone inhibited MM cell growth, and when MM cells were incubated with both pamidronate and 1.24(OH)2D2, the antiproliferative effect was enhanced [38]. In addition to in vitro research, human studies also provided promising results. Wang and colleagues investigated the relationship between 25(OH)D3 levels and motor and sensory peripheral neuropathy among MM patients who have been treated with BTZ and/or thalidomide. First, they established that 42% of patients were either 25(OH)D3 deficient (<20.0 ng/mL; 16% of patients) or insufficient (20.0–29.9 ng/mL; 26%). Moreover, the obtained results revealed that the severity of peripheral neuropathy is associated with lower vitamin D levels [41]. Eicher and colleagues found that VD deficiency was associated with poorer outcomes in lymphoma and MM patients undergoing high-dose chemotherapy followed by autologous stem cell transplantation (AHSCT) [39]. In a randomized clinical control trial conducted by Raoufinejad et al., the impact of calcitriol supplementation on short-term and long-term hematopoietic recovery, relapse-free survival (RFS) and overall survival (OS) in MM and Hodgkin’s and non-Hodgkin’s lymphoma patients undergoing high-dose chemotherapy followed by AHSCT was assessed. The obtained results revealed that recovery rates of absolute lymphocyte count (ALC) were significantly higher in the calcitriol group than in the placebo group (*p* < 0.001). On top of that, the two-year RFS was also significantly higher in the calcitriol group than in the placebo group (*p* = 0.03). However, OS was not improved by calcitriol administration in the cited trial. Nonetheless, VD supplementation appears to be a safe option that may improve RFS and ALC recovery [40]. As mentioned above, in vitro and in vivo studies delivered promising results. In addition to VD itself, there are also its synthetic analogs such as EB1089 and paricalcitol. They should be considered of particular interest due to their reduced potential to induce hypercalcemia when administered to patients. VD analogs were also tested on MM cell lines to investigate if they exhibit similar anti-cancer properties to VD. These analogs alone or in combination with other agents when added to the medium also revealed the potential to inhibit MM cell growth predominantly via induction of apoptosis and cell cycle arrest [43,49,50,51,52].

Drug resistance is one of the greatest challenges in cancer treatment. Chemotherapy is effective in reducing tumor mass and killing cancer cells while exerting tremendous selective pressure on them. As a result, in some cases, the resistant phenotype of malignant cells becomes favorable. Over time, the resistant clone may eventually prevail, and the patient becomes refractory to the therapy. There are multiple effector mechanisms and underlying molecular changes that cancer cells develop in order to achieve drug resistance. During the experiment, we managed to obtain a BTZ-resistant phenotype. We subsequently investigated the molecular alterations underlying these mechanisms. Our results revealed that the treatment of MM cells with BTZ triggered the activation of processes that increase cellular energy, such as oxidative phosphorylation (OXPHOS), the generation of precursor metabolites and energy and the ATP metabolic process, which may be crucial in the development of resistance to BTZ. The supplementation of VD and VK may limit the development of these processes.

Similar phenomena were observed in different malignancies. OXPHOS-dependent mechanisms appear to mediate drug resistance and may be a potential therapeutic target. Lee and colleagues conducted research on triple-negative breast cancer (TNBC) cells. First, they identified that drug-resistant TNBC cells exhibit co-amplification of MYC and MCL1. Since the phenotype of so-called cancer stem-like cells (CSCs) is a known feature of many chemotherapy-resistant tumors, it was then investigated whether MYC and MCL1 contributed to the enrichment of CSCs. Ultimately, they confirmed that MYC and MCL1 contribute to CSC enrichment and tumor-initiating capacity in TNBC. Next, they examined the mitochondrial respiratory capacity of the breast CSCs as well as the levels of reactive oxygen species (ROS). Upon careful analysis, the results suggested that TNBC CSCs exhibit hyperactive mitochondrial OXPHOS and in turn retain their ability to self-renew. The entire process was mediated by MYC and MCL1, and in the aftermath, HIF-1α was induced. Inhibition of HIF1-α abolishes CSC enrichment in chemotherapy-resistant TNBC. Collectively, the obtained results emphasize the role of oxidative phosphorylation in mediating drug resistance and provide a molecular background for considering HIF-1α as a therapeutic target in chemotherapy-resistant TNBC [53,54]. Similar observations regarding OXPHOS and mitochondrial function in mediating drug resistance were made in cisplatin-resistant ovarian cancer cells. The results of the study demonstrated that in cisplatin-resistant ovarian cancer cells, cisplatin activates the nuclear and mitochondrial transcription systems by upregulating PGC1α expression, which mediates the expression of mitochondrial proteins encoded by both nuclear DNA and mitochondrial DNA. This is largely due to the altered, i.e., enhanced, OXPHOS function during chemotherapy, which increases mitochondrial ROS levels. These increased ROS levels act as feedback signals from the mitochondria to the nucleus, promoting enhanced PGC1α-mediated biogenesis, thereby stabilizing mitochondrial function and allowing cancer cells to escape apoptosis [54].

Drug resistance in MM appears to be mediated in a similar manner. Matula et al. demonstrated in vitro that MM cells under the pressure exerted on them by chemotherapeutic agents tend to acquire mitochondria from autologous bone marrow stromal cells (BM-MSCs), which causes ATP levels to rise in MM cells. In parallel, mitochondrial superoxide levels decrease in MM cells. When assessing cytotoxicity in the co-culture, BM-MSCs were effective in protecting MMs against carfilzomib-induced apoptosis [55]. Another interesting study showed that the expression of genes associated with oxidative phosphorylation gradually increased with cytogenetic risk and clinical status, from monoclonal gammopathy of undetermined significance (MGUS) to relapsed and refractory MM with translocation t(4;14) [56]. Taken together, the above studies, combined with our results, suggest that cellular OXPHOS, ATP metabolism and energy generation contribute substantially to the development of the BTZ-resistant phenotype.

Cell cycle arrest and subsequent induction of apoptosis may be a promising therapeutic strategy in various cancers [57,58,59]. On the other hand, temporary cell cycle arrest, named dormancy or quiescence, is associated with drug resistance, tumor progression and metastatic potential [60]. We demonstrated that in BTZ-resistant phenotype processes related to “cell division”, “nuclear division” and “mitotic nuclear division” were significantly downregulated. Reducing the rate of cell division and slowing down the cell cycle appears to have a supposedly protective effect on MM cells. However, the exact mechanisms are yet to be determined and should be investigated in other MM cell lines as well as in ex vivo models. Nevertheless, similar observations were noted in studies investigating chemotherapy resistance in various cancers. Cole and colleagues investigated the mechanisms of cisplatin resistance in vitro using ovarian cancer cell lines. Their study revealed that the transcription regulator nuclear factor of activated T cells 4 (NFATC4) induces a quiescent state in ovarian cancer and translocates to the nucleus in response to chemotherapy. Constitutively nuclear NFATC4 is associated with a reduction in cell size and proliferation and the induction of chemotherapy resistance. NFATC4 promotes the quiescent phenotype by early downregulation of MYC [61]. In another in vitro study, tumor cells were shown to enter a reversible quiescent state in response to paclitaxel toxicity. Interestingly, the proliferative cancer cells were found to have an alkaline pH (7.3–7.5), while in the quiescent cancer cells, the pH was acidic. After subsequent analysis, it was concluded that intracellular acidification followed by protein ubiquitination has a regulatory role in quiescence entry [62]. Quiescence is a complex process that is regulated in a huge variety of ways. It was also demonstrated that epigenetic mechanisms also contribute to its pathogenesis [63]. Very little is known about dormancy in MM. Therefore, this area remains terra incognita and is worth exploring further. Although there is a scarcity of studies investigating this issue, it was established that this phenomenon also contributes to the growth and survival of MM cells. TRIM44 has been shown to be an important factor regulating the quiescence property of MM cells. TRIM44 stabilizes HIF-1α to maintain its stability, which in turn supports the survival of quiescent MM [64]. Moreover, resistance to BTZ appears to be to some extent mediated by dormancy. Cdc37, a key co-chaperone of Hsp90, is downregulated in relapsed MM, especially after BTZ treatment. This finding implies that suppression of Cdc37 induces resistance to BTZ in MM cells. Downregulation of Cdc37 or inhibition of Cdc37/Hsp90 association induces MM cell immaturity, increases quiescent MM cell populations and increases BTZ resistance in MM. Furthermore, expression of Cdc37 positively correlates with Xbp1s, a critical transcription factor for the differentiation of plasma cells in MM. Depletion/inhibition of Cdc37 downregulates Xbp1s, while overexpression of Xbp1s in MM cell lines partially reverses their immaturity and avoids resistance to BTZ [65].

Incubation with vitamins during treatment with BTZ showed inhibition and activation of “RNA processing” in MM cells. Changes in the regulation of “RNA processing” may indicate an involvement of VD in the regulation of epigenetic processes. Moreover, we identified upregulation of many genes belonging to the SNORDs family. This further strengthens the assumption about the involvement of epigenetic regulatory mechanisms, since the mentioned gene family is engaged in methylation and also regulates gene expression predominantly through post-transcriptional antisense mechanisms [66]. However, we did not identify a particular pattern of activation or inhibition of RNA-processing-related actions in the analyzed samples, which in turn suggests the complexity of epigenetic mechanisms potentially involved in the development of resistance to BTZ and requires further in-depth studies.

## 5. Conclusions

The pathophysiology of BTZ resistance is multifactorial. We showed that it is mediated by, inter alia, processes related to ATP metabolism and oxidative phosphorylation. 25(OH)D3 and VK2 MK-7, by restoring proper metabolism, may have overcome resistance to BTZ in vitro. The simultaneous supplementation of the abovementioned vitamins significantly reduced proliferation in the non-BTZ-resistant myeloma cell line as well as in the BTZ-resistant phenotype. What is more, dormancy appears to contribute to BTZ resistance as well. The demonstrated upregulation of genes from the SNORDs family suggests the involvement of epigenetic mechanisms. The discovery that VD and VK were able, at least temporarily, to overcome BTZ resistance is of paramount importance. It provides the background for further clinical trials evaluating VD and VK as potential, cheap and easy-to-implement adjuvant therapies for MM patients.

## 6. Limitations

This study is the first to show the ability of VD and VK to reduce the development of resistance to BTZ. Unfortunately, many issues remain unresolved. The study showed some potential for reducing the development of resistance to BTZ. Perhaps constant/chronic supplementation with vitamins would provide even better, long-term results. However, this is an area that requires further exploration due to the common problem of resistance in the treatment of oncological diseases. Another limitation of our study is the analysis of a single MM cell line only. Repeating our study protocol with other MM cell lines could either strengthen results that have already been obtained or shed more light on less prominent mechanisms. In addition, our analyses were conducted in vitro. It can only be assumed that similar effects would be obtained in vivo due to the complexity of interactions in the human body. The clinical application of our results should be further investigated in randomized clinical trials.

## Figures and Tables

**Table 1:** Comparison of NES values of the processes activated after second and third treatments with BTZ of U266 myeloma cells. Data compiled on the basis of GSEA analysis.

		BTZ Treatment II				BTZ Treatment III			
Symbol GO	Process Name	The Number of Genes Involved	NES	*p*-Value	*p*.adj	The Number of Genes Involved	NES	*p*-Value	*p*.adj
GO:0002181	cytoplasmic translation	136	6.098	<0.0001	<0.0001	136	5.427	<0.0001	<0.0001
GO:0009060	aerobic respiration	153	3.601	<0.0001	<0.0001	153	4.611	<0.0001	<0.0001
GO:0006119	oxidative phosphorylation	112	3.337	<0.0001	<0.0001	112	4.433	<0.0001	<0.0001
GO:0006091	generation of precursor metabolites and energy	334	3.200	<0.0001	<0.0001	334	4.164	<0.0001	<0.0001
GO:0046034	ATP metabolic process	195	3.156	<0.0001	<0.0001	195	3.956	<0.0001	<0.0001
GO:0045333	cellular respiration	182	3.080	<0.0001	<0.0001	182	4.548	<0.0001	<0.0001
GO:0022900	electron transport chain	128	2.993	<0.0001	<0.0001	128	3.653	<0.0001	<0.0001
GO:0015980	energy derivation by oxidation of organic compounds	226	2.835	<0.0001	<0.0001	226	4.117	<0.0001	<0.0001

Green indicates the NES values which increased after the third treatment with BTZ in relation to the second.

## Data Availability

The data presented in this study are available upon request from the corresponding author.

## Supplementary Materials

The following supporting information can be downloaded at: https://www.mdpi.com/article/10.3390/nu14235190/s1, Table S1: Involvement of genes from the SNORDs group in the development of resistance to BTZ in U266 myeloma cells. Data compiled on the basis of DAVID analysis.
