# Supplementary material for: The Evidence That 25(OH)D3 and VK2 MK-7 Vitamins Influence the Proliferative Potential and Gene Expression Profiles of Multiple Myeloma Cells and the Development of Resistance to Bortezomib
Source: Nutrients. 2022 Dec 6;14(23):5190. doi: 10.3390/nu14235190 (PMC9736786; doi:10.3390/nu14235190)
Supplement: Supplementary file 1 [file nutrients-14-05190-s001.zip › nutrients-2014332-supplementary.pdf]

Table S1. Involvement of genes from the SNORDs group in the development of resistance to BTZ in U266 myeloma cells. Data compiled on the basis of DAVID analysis.

**BTZ treatment I**		**BTZ treatment II**		**BTZ treatment III**	
up	down	up	down	up	down
-	SNORD19B, SNORA68, SNORD51, SNORD42B, SNORD37, SNORD33, SNORD32A, SNORA10, SNORD21, SNORA52, SNORA75, SNORA55, SNORA50C, SNORD1C, SNORD50B, SNORD19, SNORD69, SNORD38B	SNORD36B, SNORD18A, SNORA26, SNORA78, SNORD23, SNORD94	SNORD116-20, SNORA80B, SNORD15B, SNORD12C, SNORA71A, SNORD63, SNORD60, SNORD51, SNORD43, SNORD109A, SNORD95, SCARNA22, SNORA1, SNORA5B, SNORA22, SNORA51, SNORA55, SNORA71C, SNORA81, SNORD1C, SNORD12, SNORD45C, SNORD99, SNORD97, SNORD3A, SNORD14C, SNORD27	SNORD12B, SNORD102, SNORA71A, SNORD63, SNORD32A, SNORA10, SNORD16, SNORD74, SNORA21, SNORA5C, SNORA71C	SNORD116-4, SNORD116-20, SNORD58C, SNORA80B, SNORA66, SNORD59A, SNORD54, SNORD47, SNORD42B, SNORD35A, SNORD101, SNORD100, SNORD20, SNORA41, SNORA48, SNORA13, SCARNA22, SNORA5B, SNORA24, SNORA51, SNORA81, SNORA25, SNORD12, SNORD5, SNORD99, SNORD97, SNORD14C, SNORD29, SNORD30, SNORD27
